# Supplementary material for: Cell-Free Tumor DNA Detection-Based Liquid Biopsy of Plasma and Bile in Patients with Various Pancreatic Neoplasms
Source: Biomedicines. 2024 Jan 18;12(1):220. doi: 10.3390/biomedicines12010220 (PMC10813046; doi:10.3390/biomedicines12010220)
Supplement: Supplementary file 1 [file biomedicines-12-00220-s001.zip › Figure S1.pdf]

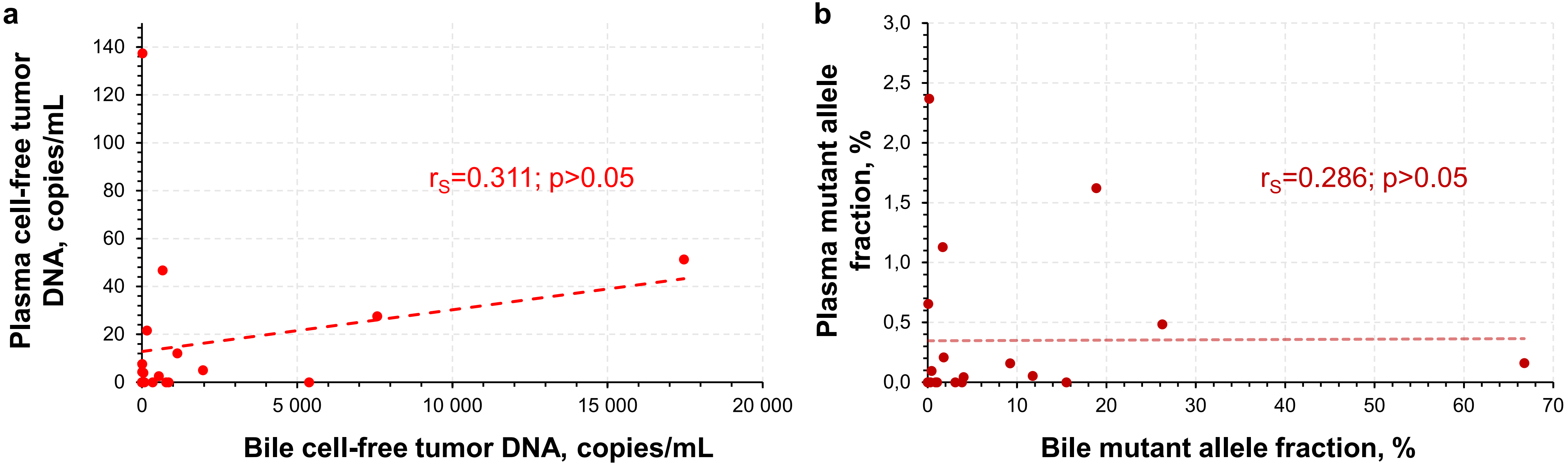

Figure S1. Correlation of cell-free DNA analysis results for plasma and bile in patients with pancreatic ductal adenocarcinoma. **(a)** Scatter plot for data presented as copies per 1 mL of biomaterial. **(b)** Scatter plot for data presented as tumor DNA fraction. Cell-free tumor DNA was detected using digital droplet polymerase chain reaction on a QX200 AutoDG ddPCR System (Bio-Rad Laboratories, Inc) based on presence of common KRAS mutations in G12/G13, Q61 hotspots. r_S – Spearman's rank correlation coefficient.
