# Supplementary material for: Cell-Free Tumor DNA Detection-Based Liquid Biopsy of Plasma and Bile in Patients with Various Pancreatic Neoplasms
Source: Biomedicines. 2024 Jan 18;12(1):220. doi: 10.3390/biomedicines12010220 (PMC10813046; doi:10.3390/biomedicines12010220)
Supplement: Supplementary file 1 [file biomedicines-12-00220-s001.zip › Figure S2.pdf]

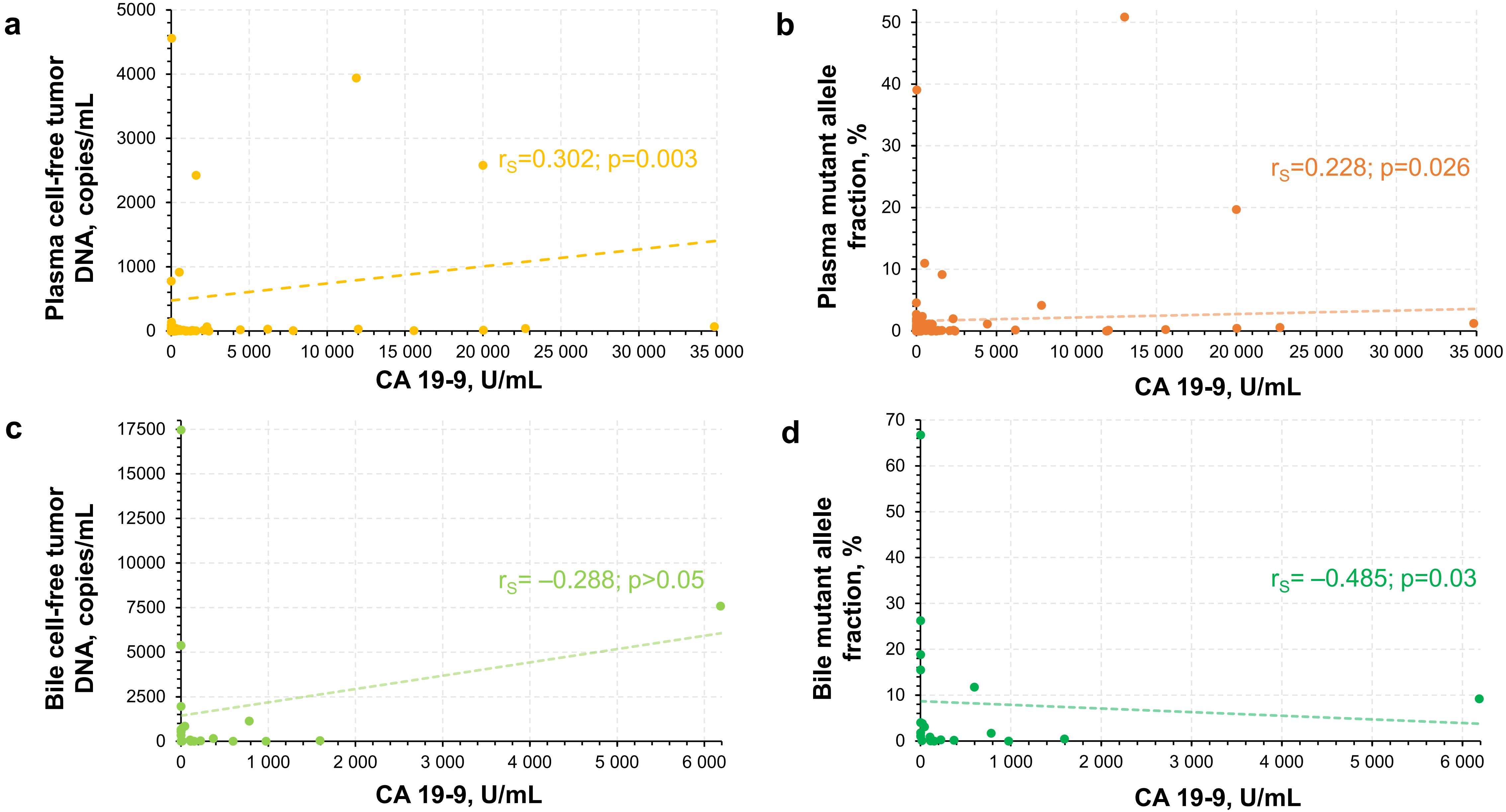

Figure S2. Correlation of cell-free DNA analysis results with serum CA 19-9 levels in patients with pancreatic ductal adenocarcinoma. **(a)** Scatter plot for cell-free tumor DNA concentration (copies per 1 mL of biomaterial) in plasma (an outlier with a cell-free tumor DNA concentration of 36 840 copies/mL and an outlier with a CA 19-9 level of 120 000 U/mL were excluded in order to preserve the proportions of the plot). **(b)** Scatter plot for mutant allele fraction in plasma. **(c)** Scatter plot for cell-free tumor DNA concentration in bile (copies per 1 mL of biomaterial). **(d)** Scatter plot for mutant allele fraction in bile. Cell-free tumor DNA was detected using digital droplet polymerase chain reaction on a QX200 AutoDG ddPCR System (Bio-Rad Laboratories, Inc) based on presence of common KRAS mutations in G12, G13, Q61 hotspots. r_S – Spearman's rank correlation coefficient.
