# Supplementary material for: Cell-Free Tumor DNA Detection-Based Liquid Biopsy of Plasma and Bile in Patients with Various Pancreatic Neoplasms
Source: Biomedicines. 2024 Jan 18;12(1):220. doi: 10.3390/biomedicines12010220 (PMC10813046; doi:10.3390/biomedicines12010220)
Supplement: Supplementary file 1 [file biomedicines-12-00220-s001.zip › Figure S3.pdf]

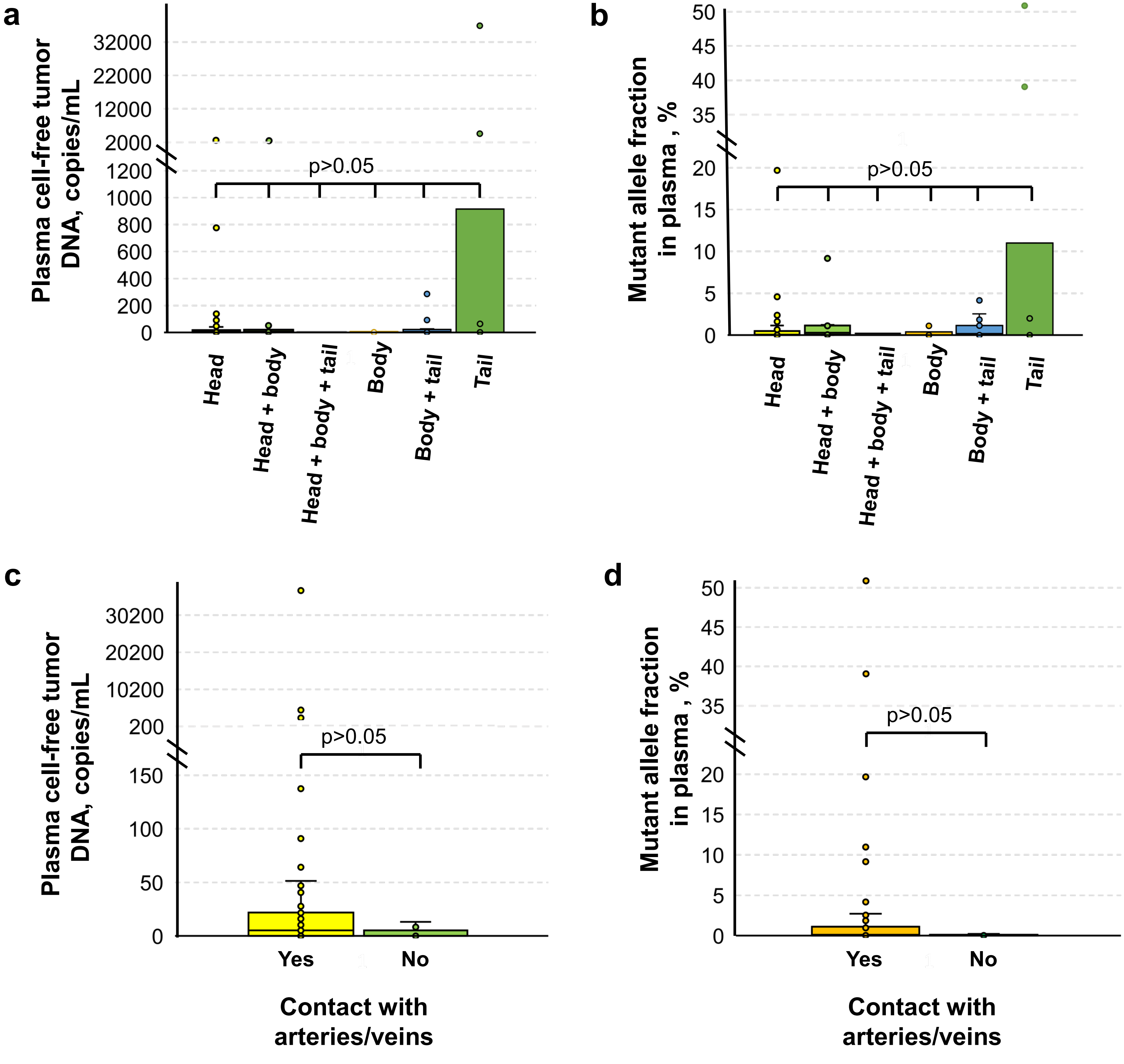
a
Plasma cell-free tumor DNA, copies/mL
32000
22000
12000
2000
1200
1000
800
600
400
200
0
p>0.05
Head
Head + body
Head + body + tail
Body
Body + tail
Tail
b
Mutant allele fraction in plasma , %
50
45
40
35
20
15
10
5
0
p>0.05
Head
Head + body
Head + body + tail
Body
Body + tail
Tail
c
Plasma cell-free tumor DNA, copies/mL
30200
20200
10200
200
150
100
50
0
p>0.05
Yes
No
Contact with arteries/veins
d
Mutant allele fraction in plasma , %
50
45
40
35
20
15
10
5
0
p>0.05
Yes
No
Contact with arteries/veins

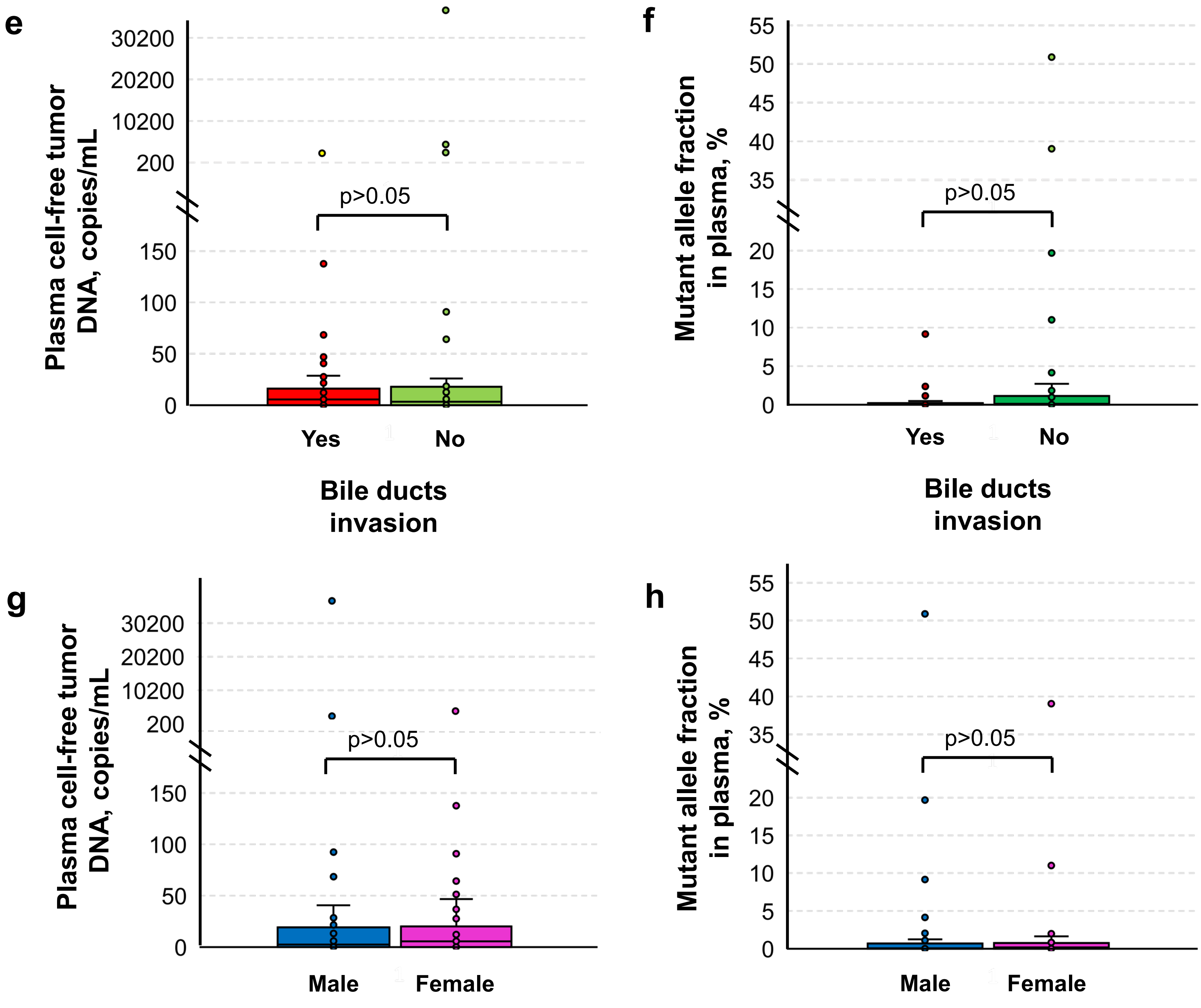

e
Plasma cell-free tumor DNA, copies/mL
30200
20200
10200
200
150
100
50
0
p>0.05
Yes
No
Bile ducts invasion
f
Mutant allele fraction in plasma, %
55
50
45
40
35
20
15
10
5
0
p>0.05
Yes
No
Bile ducts invasion
g
Plasma cell-free tumor DNA, copies/mL
30200
20200
10200
200
150
100
50
0
p>0.05
Male
Female
h
Mutant allele fraction in plasma, %
55
50
45
40
35
20
15
10
5
0
p>0.05
Male
Female

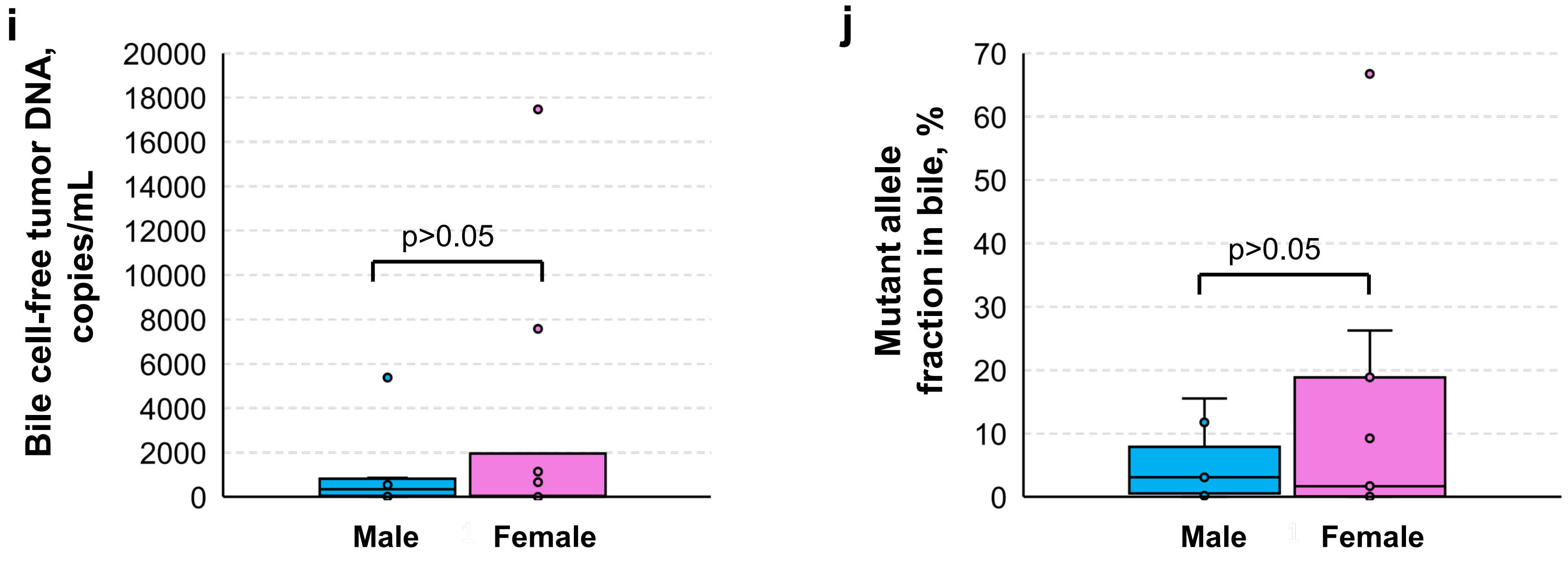


**Figure S3.** Comparison of cell-free tumor DNA analysis results in patients with various localization characteristics of the tumor and sex. **(a)** Box plot for data presented as copies per 1 mL of plasma (localization of the tumor in pancreas). **(b)** Box plot for data presented as mutant allele fraction in plasma (localization of the tumor in pancreas). **(c)** Box plot for data presented as copies per 1 mL of plasma (contact of the tumor with arteries/veins). **(d)** Box plot for data presented as mutant allele fraction in plasma (contact of the tumor with arteries/veins). **(e)** Box plot for data presented as copies per 1 mL of plasma (invasion of the tumor into bile ducts). **(f)** Box plot for data presented as mutant allele fractionin plasma (invasion of the tumor into bile ducts). **(g)** Box plot for data presented as copies per 1 mL of plasma (sex). **(h)** Box plot for data presented as mutant allele fraction in plasma (sex). **(i)** Box plot for data presented as copies per 1 mL of bile (sex). **(j)** Box plot for data presented as mutant allele fraction in bile (sex).

Comparison of bile cell-free tumor DNA analysis results in patients with various localization characteristics of the tumor was not visualized due to certain characteristics being predominant in patients who donated bile (localization of the tumor in the head of pancreas, positivity for contact with arteries/veins and invasion into bile ducts).

Cell-free tumor DNA was detected using digital droplet polymerase chain reaction on a QX200 AutoDG ddPCR System (Bio-Rad Laboratories, Inc., Hercules, CA, USA) based on presence of common *KRAS* mutations in G12/G13, Q61 hotspots. Determination of localization characteristics of the tumor was carried out using computed tomography, magnetic resonance imaging, and positron emission tomography/computed tomography with 18FDG according to medical indications.
